# Supplementary material for: Assessment of Carbon Substrate Catabolism Pattern and Functional Metabolic Pathway for Microbiota of Limestone Caves
Source: Microorganisms. 2021 Aug 23;9(8):1789. doi: 10.3390/microorganisms9081789 (PMC8398112; doi:10.3390/microorganisms9081789)
Supplement: Supplementary file 1 [file microorganisms-09-01789-s001.zip › microorganisms-1334432-supplementary.pdf]

Supplementary tables and figures

Supplementary Table S1. Details of sampling environmental conditions

Sample ID	Sample type	Detection time	Ambient temperature	Humidity	Sunlight (LUX)	Soil temperature	Sampling zone
LR-01	Rock	10:44 AM	21.5	60 %	256-260	Nil	Outside cave
LS-01	Soil				256-261	18.5	
LR-05	Rock	11:55 AM	23.3	63 %	0	Nil	Inside cave
LS-05	Soil				0	18.3	

LR-01, LS-01: outside the cave; LR-05, LS-05: inside the cave

Supplementary Table S2. Pearson's correlation test against predominant bacteria phylum with environmental factors

Correlations

		1	2	3	4	5	6	7	8	9	10	11
1	**Planctomycetes**											
2	**Proteobacteria**	0.86										
3	**Acidobacteria**	-0.57	-0.87									
4	**Actinobacteria**	-0.71	-0.71	0.30								
5	**Nitrospirae**	0.07	-0.03	0.46	-0.68							
6	**Chloroflexi**	-0.89	-0.86	0.80	0.39	0.37						
7	**Gemmatimonadetes**	-0.83	-0.86	0.86	0.32	0.46	.99**					
8	**Cyanobacteria**	0.77	.98*	-0.95	-0.58	-0.19	-0.85	-0.88				
9	**Temperature**	0.13	-0.38	0.52	0.28	-0.08	-0.06	0.03	-0.46			
10	**Humidity**	-0.91	-0.89	0.54	0.93	-0.38	0.70	0.64	-0.78	0.19		
11	**Sunlight**	0.91	0.89	-0.54	-0.93	0.39	-0.70	-0.64	0.78	-0.19	-1.00**	

*. Correlation is significant at the 0.05 level (2-tailed).

**. Correlation is significant at the 0.01 level (2-tailed).

Supplementary Table S3. KEGG pathway normalized reads for rock and soil samples collected outside and inside the cave.

Super pathway	Sub pathway	LR-01	LS-01	LR-05	LS-05
Carbohydrate metabolism	Starch and sucrose metabolism	0.0051	0.0082	0.0040	0.0062
	Galactose metabolism	0.0845	0.0731	0.0817	0.0809
	Amino sugar and nucleotide sugar metabolism	0.0004	0.0010	0.0004	0.0005
	Fructose and mannose metabolism	0.0003	0.0000	0.0000	0.0000
	Pentose phosphate pathway	0.0288	0.0162	0.0245	0.0245
	Citrate cycle (TCA cycle)	0.0420	0.0746	0.0685	0.0543
	Ascorbate and aldarate metabolism	0.0808	0.0700	0.0802	0.0791
Amino acid metabolism	Arginine and proline metabolism	0.1093	0.0798	0.1075	0.1069
	Phenylalanine metabolism	0.0006	0.0021	0.0041	0.0014
	Glycine, serine and threonine metabolism	0.0499	0.0324	0.0368	0.0418
Other amino acid metabolism	Glutathione metabolism	0.0014	0.0061	0.0045	0.0044

**Lipid metabolism**	**Glycerophospholipid metabolism**	0.0047	0.0126	0.0037	0.0071
**Xenobiotics biodegradation and metabolism**	**Bisphenol degradation**	0.0382	0.0321	0.0299	0.0359
	**Benzoate degradation**	0.0139	0.0163	0.0134	0.0136
**Energy metabolism**	**Photosynthesis - antenna proteins**	0.0000	0.0000	0.0001	0.0000
	**Carbon fixation pathways in prokaryotes**	0.0027	0.0071	0.0044	0.0035
	**Methane metabolism**	0.0101	0.0135	0.0092	0.0054
**Membrane transport**	**ABC transporters**	0.0319	0.0507	0.0282	0.0216

LR-01, LS-01: outside the cave; LR-05, LS-05: inside the cave

Supplementary Table S4. Primer information list.

Primer type	Primer name	Base pair sequence
Forward	341F	CCTACGGGNGGCWGCAG
Reverse	805R	GACTACHVGGGTATCTAATCC

Supplementary Table S5. Microbial community beta diversity index.

**LR-01**	**LS-01**	**LR-05**	**LS-05**
Microbial community beta diversity			
**D**	**D**	**D**	**D**
0.87	0.85	0.84	0.86

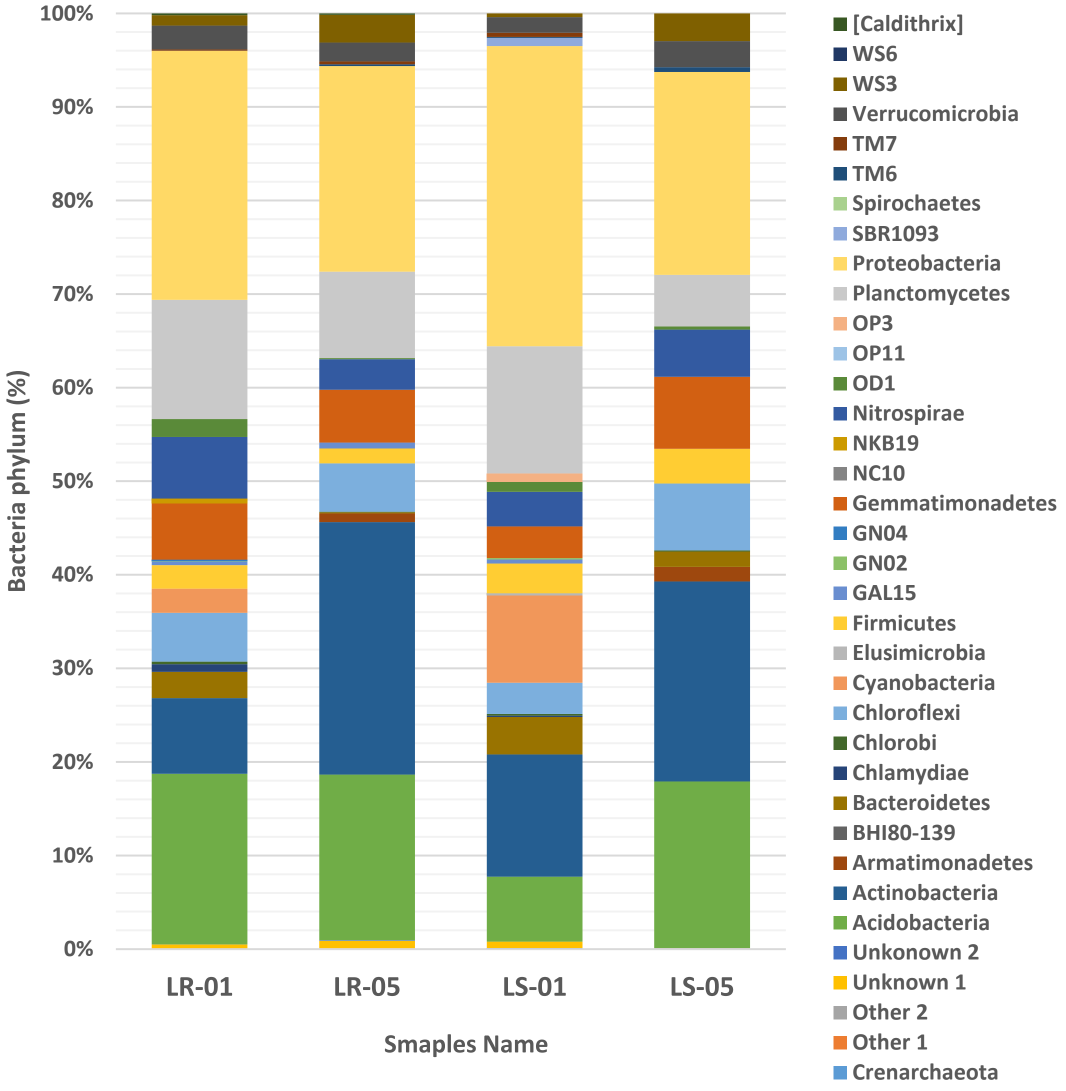

Supplementary Figure S1. Abundance of bacteria phylum in all rock and samples

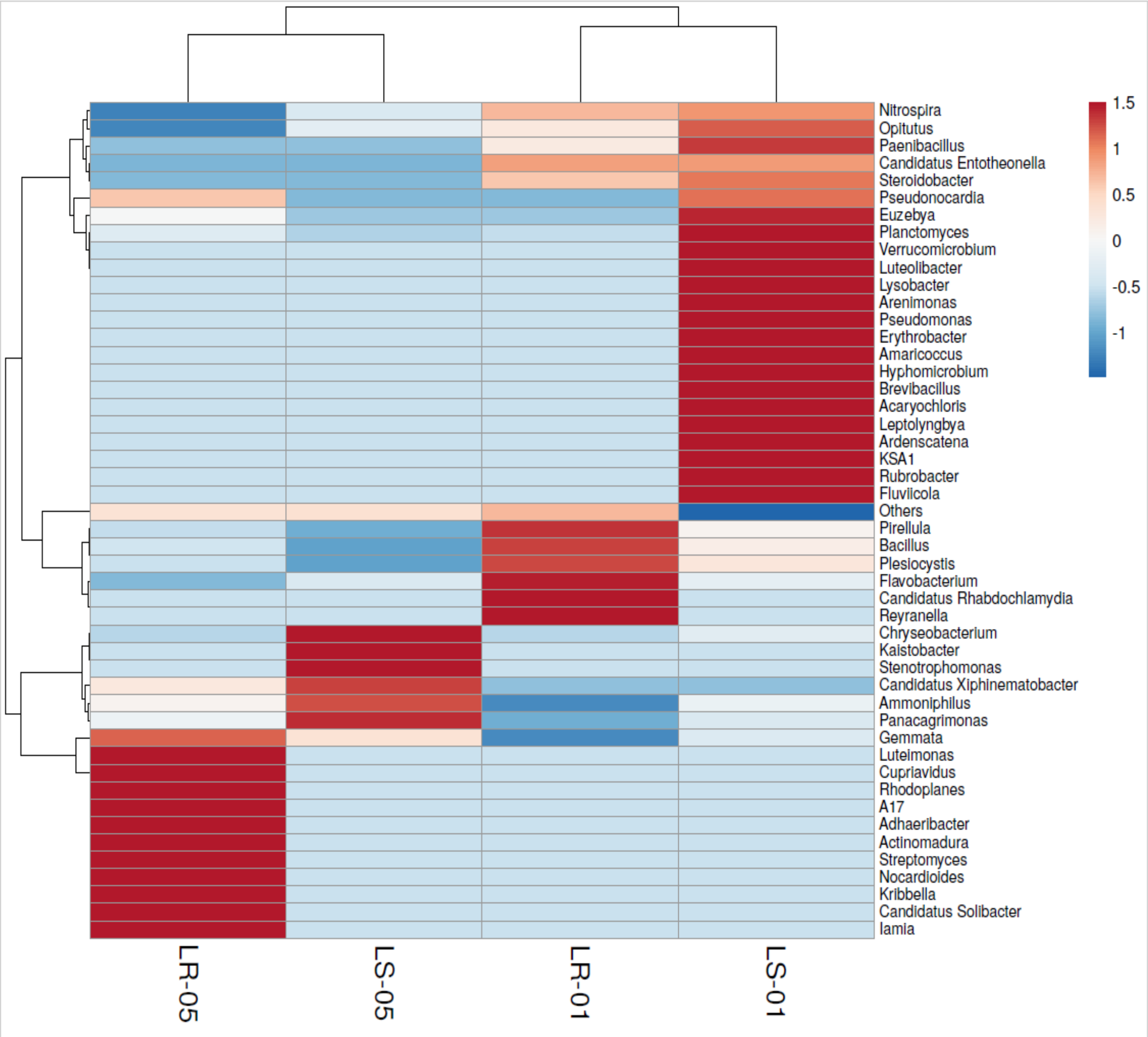

Supplementary Figure S2. Heatmap analysis with dendrogram plot at the genus level for all samples
